# An intersectional gender analysis of familial and socio-cultural drivers of inequitable scientific career progression of researchers in Sub-Saharan Africa

**DOI:** 10.1186/s41256-021-00213-3

**Published:** 2021-08-17

**Authors:** Millicent L. Liani, Isaac K. Nyamongo, Justin Pulford, Rachel Tolhurst

**Affiliations:** 1 Department of International Public Health, Liverpool School of Tropical Medicine, Pembroke Place, Liverpool, L3 5QA UK; 2 Faculty of Health and Life Sciences, University of Liverpool, Liverpool, L69 3BX UK; 3 Division of Research and Innovation, The Cooperative University of Kenya, Nairobi, Kenya

**Keywords:** Researchers’ lived experiences, Socio-cultural influences, Family, Gender equity, Intersectional gender analysis, Scientific career progression, Sub-Saharan Africa, Social power relations, Research capacity strengthening

## Abstract

**Background:**

Sub-Saharan Africa (SSA) suffers from a dearth of concrete information on the causes of women’s under-representation in scientific research workforce particularly at higher levels compared with the wealth of information that exists in the global north. The goal of this study was to illuminate familial and socio-cultural drivers that contribute to intersectional gender inequities in scientific career progression in SSA to inform strategies that could promote career equity for African scientific researchers.

**Methods:**

This study was nested within the context of ‘Developing Excellence in Leadership, Training and Science in Africa’ (DELTAS Africa)—a health-based scientific research capacity strengthening initiative. It adopted an exploratory qualitative cross-sectional study design. In-depth interviews were conducted among 58 (32 Female and 26 Male) trainees/research fellows at various career stages, affiliated to three purposively selected African Research Consortia. The interviews were conducted between May and December 2018 in English. The data were analysed inductively based on emergent themes.

**Results:**

The study participants were nationals of thirteen SSA countries. More female than male participants had young children. Four themes were identified. They illustrate women’s and men’s characterisation of the normative career pathway and progression requirements which calls for significant ‘time’ commitments (theme 1), and how social power relations of gender within the family and wider society shapes their participation in scientific research activities (theme 2). This culminates in researchers'' differential experiences of navigating between the ‘two different lives’—family and career, and the resultant implications for their career progression and personal well-being (theme 3). Women researchers made different and conscious trade-offs for navigating the ‘two different lives’ by utilising various metaphors such as the ‘biological clock and career clock’, the ‘glass ball and rubber ball’, and the concept of ‘sacrifice’ (theme 4).

**Conclusions:**

This study is the first of its kind to demonstrate how intersectional gender analysis through use of qualitative research methods may provide novel insights into the hidden familial and socio-cultural drivers of gender inequitable scientific research career progression. It offers important policy and practice measures and approaches for fostering career equity for women and men scientists within research capacity strengthening initiatives in SSA.

## Background

Women’s under-representation in scientific careers, and especially in senior positions is a well-known and persistent global problem [1–3]. A commonly shared explanation is that women experience conflict in balancing scientific work and familial responsibilities (i.e. [1, 4–10]), thereby leaving them with less time for the former [11]. Existing literature on gender and science careers in industrialised countries shows that women’s slow progression and attrition at each stage of the scientific career ladder is due to career processes that are influenced by multifaceted social forces at individual, familial, and societal levels [12]. Such impediments to women’s career progression tend to be more pronounced in low and middle income countries [13], although there is also substantial variation across contexts due to religious, socio-cultural, economic and political differences among others. For example, studies from South and South-East Asia have pointed to the influence of religious and socio-cultural norms, values and traditions, which allocate gender roles to men as family breadwinners and women as caregivers [13, 14]. Such gendered division of labour has been identified as promoting notions of ‘ideal’ women as dutiful wives, mothers and homemakers, who are expected to take more family responsibilities than men [15],  constraining their opportunities in the workplace [14]. In majority of Islamic contexts in particular, restrictions on women’s mobility and participation in employment, including the association of women working as a threat to family honour, have been identified as limiting women’s career opportunities and progression [14, 16].

Extant literature from Sub-Saharan Africa (SSA) indicates that family obligations affect women differently from men, as women spend on average more time on caring for children and the elderly [1]. Nonetheless, the distinct factor for African women research scientists is they are more likely to be married with families in comparison to their European or North American counterparts at the same stage in their careers [4]. Indeed, in most African societies, men are privileged as they are not expected to contribute to domestic labour and childcare, resulting in women’s constraint of juggling marriage and family life while pursuing a scientific career [4]. However, in SSA, there is a dearth of concrete information on the causes of women’s under-representation in scientific research workforce particularly at higher levels [17], compared with the wealth of information that exists in the global north [18, 19]. In addition, SSA still suffers from a paucity of empirical studies about how gender intersects with other individual multiple social identities to produce inequities in career progression outcomes for both women and men research scientists, as most of the available studies have focused only on women as a homogenous group [20]. This presents a knowledge gap about the career experiences of women and men scientists in the SSA [7, 21].

In this paper, we illuminate familial and socio-cultural drivers that contribute to intersectional gender inequities in scientific career progression in SSA by drawing on lived experiences of women and men researchers. The data presented are part of a wider research study set within the context of ‘Developing Excellence in Leadership, Training and Science in Africa’ (DELTAS Africa)—a health-based scientific research capacity strengthening initiative. This is a 5-year (2015–2020) initiative whose vision is to train and develop the next generation of internationally competitive African scientific health researchers and research leaders while fostering career pathways [22]. The ultimate goal of this study was to produce evidence from a holistic, gender comparative and intersectional perspective that can be used to develop strategies to promote career equity for internationally competitive African scientific researchers.

## Methods

### Theoretical and conceptual framing

**Fig. 1:**
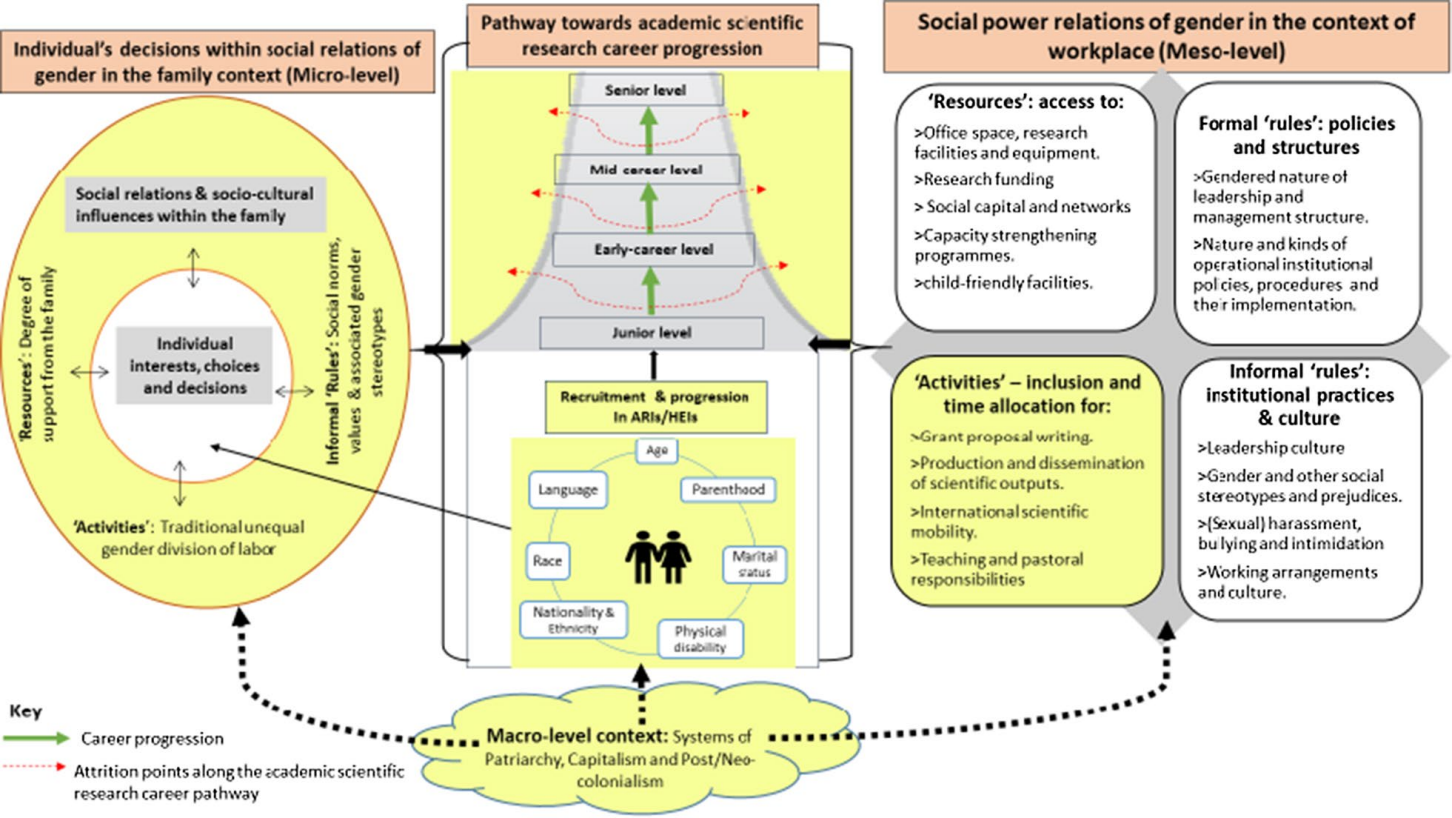
A conceptual framework for understanding intersecting gender inequities in academic scientific career progression in SSA

The empirical research for this study was informed by three theories and models: Systems of Career Influences Model [23], Social Relations Approach [24–26], and Intersectionality theory [27, 28]. These three theoretical and conceptual models were drawn together to form an integrated conceptual framework [20] which was developed based on existing evidence around the current research problem within the context of SSA as presented in Fig. 1 below.

The Systems of Career Influences Model [23] provides the central core of the framework, focusing on the interplay between socio-cultural influences within the family and organizational factors in shaping career advancement of women. Kabeer’s Social Relations Approach [24] provides key dimensions for an institutional gender analysis – within the family and workplace, expressed as ‘rules’ (formal and informal), ‘resources’ and ‘activities’, which are all permeated by ‘power’. The intersectionality lens [27, 28] is then explicitly added to highlight the multiple social identities and related power of these individuals according to aspects such as age, professional cadre, marital status, ethnicity, language minority, (dis)ability, and parenthood.

We used this integrated conceptual framework as a lens for understanding how gendered social relations and processes within the institution of the family interact with societal norms, values and expectations in shaping career progression opportunities of women and men in their day-to-day scientific research ‘activities’. We have taken gender as a key entry point into analysing the positionality and experiences of individual researchers, who according to an intersectionality perspective, may further be identified as (dis)advantaged based on other multiple intersecting social categories. Individuals may either get ‘stuck’ at a relatively junior level or opt out of the scientific career path. In this paper, as indicated in the components of the framework highlighted in yellow, we focus on how participation in scientific research activities (right box-lower level), is influenced by the social relations of gender within the family context—micro-level system (left box)—which determines progression along the pathway towards academic scientific career ladder for women and men scientific researchers in SSA, who have multiple social identities (middle box). We also indicate how such processes are reinforced by the macro-level systems of patriarchy, capitalism, and neo/colonialism in producing and reproducing inequities. The remaining constituents of the framework are explored in another paper focused on the gendered nature of the social power relations of the workplace [29].

### Study design and setting

We adopted an exploratory qualitative cross-sectional study design. The research was conducted within the context of the DELTAS Africa initiative. The initiative is coordinated by the African Academy of Sciences’ Alliance for Accelerating Excellence in Science in Africa,^1^ and implemented by a network of eleven African-led health research capacity strengthening programmes, commonly referred to as DELTAS Africa Research Consortia (DELTAS ARC). The DELTAS ARC offers collaborative research training programmes in various scientific disciplines spanning 54 lead and partner institutions (research organizations and universities) across SSA, in partnership with Northern academic institutions. In doing so, it facilitates career development of postgraduate science students (Masters and Doctorate), who are referred to in this study as junior researchers, and scientific research professionals (post-doctoral fellows and mid-level researchers), who pursue research work/studies at institutions in their home- or other African-countries.

### Study population and sampling strategy

The study population comprised of all women and men research fellows and scientists who were affiliated with or working within the purposively selected DELTAS ARC. The unit of analysis is individual women and men research fellows and scientists who were integrated within selected DELTAS ARC. The findings could be generalisable to similar populations elsewhere, although this should be done with caution in unrelated contexts or settings.

We adopted the principles of maximum variation sampling. This allowed us to discover patterns for core elements or dimensions that hold across a diverse sample, as well as unique or distinctive variations [30]. We used a two-tiered purposive sampling strategy for selection of: (1) consortia and (2) participants within the sampled consortia. Step one involved purposive sampling of three DELTAS ARC. These were selected on the basis of: regional representation in SSA (Eastern, Southern, and West and Central Africa); representation of consortia that are located in English and French speaking countries; presence of fellows of diverse nationalities recruited from different African countries; and presence of fellows at various career stages including Masters (MSc), doctoral (PhD), post-doctoral research fellows (PDF) and mid-career research (MCR) scientists.

For step two, we sought heterogeneity within each of the purposively sampled DELTAS ARC by using gender as a primary selection criterion for in-depth interview (IDI) study participants. Other dimensions of multiple social identities were sought along axes of career stage, scientific discipline, duration in the programme/institution, and nationality. A list containing such information was provided by the research directors of the sampled ARC, which aided in purposive selection of study participants.

### Data collection

Data were collected by using in-depth interviews with trainees/research fellows at various career stages affiliated to the DELTAS ARC. Interviews aimed at exploring qualitative narratives about their lived experiences in undertaking scientific research activities and the familial and societal expectations and relations shaping their career progression. We collected additional information about personal identities such as age, marital status, presence of children, nature of partnership (dual or non-dual career couple), through administering a brief questionnaire before commencing IDIs. During the interviews, we asked the participants to reflect on how such identities shaped their everyday experiences in their scientific career taken.

In total, 58 participants were interviewed, of whom 32 were female and 26 were male, across the three selected ARC. Most interviews (n = 47/58) were conducted in-person by the lead author (ML), a social science doctoral candidate with extensive experience in conducting qualitative interviews, at consortia secretariat or annual scientific meetings. The remainders were conducted via skype and telephone. Interviews were conducted between May and December 2018, all in English. Despite making provision for assistance to conduct some interviews in French, all the Francophone study participants expressed that they were comfortable conversing in English. All interviews were audio-recorded using a digital dictaphone, alongside note taking. On average, the interviews lasted 90 min.

### Data processing and analysis

Quantitative data on participants’ personal identities were analysed descriptively using  Statistical Package for Social Sciences version 25. All audio data were transcribed verbatim by an experienced qualitative research assistant. The transcripts were verified by comparing the audio files and scripts with the field notes. Once this process was complete, transcripts were sent to respective study participants for member-checking to ensure their views were appropriately captured. This process also allowed the participants to identify content they preferred to be anonymised e.g. individual characteristics and statements that could identify them.

Following member checking, the majority of participants asked to have the identities of their ARC and affiliated institution, number of children, country of origin, disciplinary field of study withheld for confidentiality purposes. In addition, they suggested that findings be presented as views and experiences of participating DELTAS Africa research fellows as a whole. All anonymised identifiers have been simplified accordingly. However, to enable presentation of an intersectional gender analysis, other identities such as age (provided in range), marital status, nature of partnership, and presence of dependents are anonymously presented where necessary. ML organised and coded the data in QSR International’s NVivo 11 qualitative data management software, and analysed these inductively based on emergent themes, whilst aligning the themes to the developed integrated conceptual framework for understanding intersecting gender inequities in academic scientific career progression in SSA [20]. ML utilised a grounded theory approach, employing constant comparative analysis [31, 32]. All illustrative quotes have been carefully reviewed for their potential to reveal individuals’ identity.

## Results

This section presents findings on characteristics of the study participants, as well as the four interrelated themes that were identified. Specifically, the themes illustrate how women’s and men’s participation in scientific research activities is shaped by interactions between familial and socio-cultural drivers, and the structure of normative career pathways. In this process, gender intersects with other aspects of identities, leading to different working experiences and inequities in career progression.

### Characteristics of the sample

The study participants were nationals of thirteen SSA countries across Eastern (Uganda, Kenya, Rwanda, Somali), Southern (Zambia, Botswana and South Africa), and West and Central Africa (Senegal, Ghana, Nigeria, Benin, Mali and Cameroon). The majority identified English as their everyday language of scientific communication (52/58) while the rest reported French. Regardless of gender, most study participants were from less educated family backgrounds (46/58), where no parents or siblings had attended university. More female than male participants had young children and the women at early career stages were more likely to have young children than men. Table 1 summarises the general socio-demographic characteristics of the study participants.

**Table 1:** Socio-demographic characteristics of the study participants (n = 58)

Gender		Other characteristics	Total (n = 58)	Msc (n = 14)	PhD (n = 19)	PDF (n = 18)	MCR (n = 7)
Women (n = 32)	Age range	25–29	9	7	2	–	–
		30–34	12	2	9	1	–
		35–39	5	–	–	2	3
		40–44	4	–	1	2	1
		45–49	2	–	–	1	1
		Total	32	9	12	6	5
	Marital status	Unmarried*	16	7	4	3	2
		Married	16	2	8	3	3
		Total	32	9	12	6	5
	With children < 5 years	Unmarried (16)	4/16	0/7	0/4	2/3	2/2
		Married (16)	12/16	2/2	6/8	3/3	1/3
		Total (32)	16/32	2/9	6/12	5/6	3/5
	Family educational Background**	High education	8	2	2	1	3
		Low education	24	7	10	5	2
		Total	32	9	12	6	5
Men (n = 26)	Age range	25–29	4	3	1	–	–
		30–34	8	2	3	3	–
		35–39	9	–	3	5	1
		40–44	2	–	–	2	–
		45–49	3	–	–	2	1
		Total	26	5	7	12	2
	Marital status	Unmarried*	11	5	4	1	1
		Married	15	–	3	11	1
		Total	26	5	7	12	2
	With children < 5 years	Unmarried (11)	0/11	0/5	0/4	0/1	0/1
		Married (15)	11/15	0	1/3	10/11	0/1
		Total (26)	11/26	0/5	1/7	10/12	0/2
	Family educational Background**	High education	4	1	1	2	0
		Low education	22	4	6	10	2
		Total	26	5	7	12	2

^*^ ‘Unmarried’ includes those identifying as single (never married), divorced, or separated, all grouped together to increase anonymity and confidentiality

^**^High education = parent or sibling has university-level education, otherwise classified as low education

A further analysis of the type of partnership as it relates to scientific research professional career for all married female and male participants showed that for the majority of relationships (24/31), only one partner of the study participants, was in a such a profession.^2^ For the remainder of married study participants (7/31), both partners were in scientific research professional careers, referred to as dual scientific career couples, and the majority of these participants were women (5/7). Notably, both female and male participants in dual scientific career unions were at PhD (4) and PDF (3) career stage, and nearly all of them (6/7) had under five-year-old children.

### Theme 1: Normative career pathway and progression requirements

Overall, study participants consistently described a scientific research career as beginning with a postgraduate degree, followed by post-doctoral research experience, mainly through working in a research group on a grant led by a senior researcher. Thereafter, researchers are expected to obtain their own independent research grant to establish a research group and demonstrate leadership through running and managing it. Progression from one scientific career stage to the next calls for significant ‘time’ commitments, outside of core working hours, requiring:

…Long working hours with a lot of lab and fieldwork, grant proposal writing, production of scientific outputs…[and] …international scientific mobility that requires being ready to pack and go at any time (IDI, Male, #14, MCR).

The latter aspect of availability for short-term travel to spend research time in another context, was perceived as instrumental for enabling career progression.^3^ This was through gaining scientific research skills, enhancing visibility through presentation in scientific fora and developing and fostering the professional networking and research collaborations that are key to obtaining research grants.

### Theme 2: Social power relations of gender within the family and wider society

Participants’ narratives elucidated the ways that social power relations of gender shaped their struggle in meeting the career progression requirements, specifically, the unequal gender division of labour within the family, and informal ‘rules’ of gendered social norms, values and stereotypes in society.

#### Unequal gender division of labour within the family

Scientific research career progression requirements were reported as more challenging for women compared with men. Most female and male participants consistently identified competing expectations of career and family responsibilities as a factor impeding research productivity, and agreed that this is particularly the case for women. A common notion was that ‘in Africa’, women’s key role is to perform domestic chores and meet marital and family obligations, while men are expected to be ‘breadwinners’ as exemplified in the quote below:

Women especially in Africa are at a more disadvantaged point because a woman is central in the family obligations especially when children are young. Men are expected to go out there toiling to get money for the family as breadwinners (IDI, Female, #01, PhD).

Fulfilment of such gendered responsibilities entails normative symbolic requirements of constant ‘availability’ and visible prioritisation of family and marriage. For women, this included an ideal of always being at home to fulfil domestic responsibilities such as caring roles for children, and other family members such as siblings and elderly parents, as well as participation in family events. For men, it involved being at home to participate and sometimes preside over customary family obligations, and safeguard their families and marriages, in addition to fulfilling their breadwinning roles as household heads.

#### Informal ‘rules’: gendered social norms and values of marriage and childbearing

The influence of informal ‘rules’ characterised by gendered social norms and values, and socially ascribed gender roles, responsibilities, and expectations was common in most female and some male participant narratives at all career stages. This was mediated by other aspects of individual identities such as age, marital status, parental status, religion, positional hierarchy in the family, and social norms around birth order, which presented differential career advancement challenges.

A common struggle for some unmarried female researchers at all career stages was conformity to societal values which stress the centrality of marriage and motherhood for women. Some experienced pressure from their parents, extended family members and religious leaders to get married and have children, at a time when their peers were establishing their science career. The timing of this depended on the contextually specific societal expected age at marriage for women and men. Most participants asserted that in some social contexts with strong religious beliefs and values around marriage, women were expected to either get married in their early, mid or late twenties, regardless of education, and start bearing children not later than three years after marriage. In contrast, men were required to marry either in their mid-to-late twenties or early thirties. Some unmarried women who defied such societal expectations occasionally received cautionary statements such as “*your eggs will die”* (IDI, Female, #09, MSc) or “ *I am getting old…I need to see your child before I die”* (IDI, Female, #07, MSc). Such emotional pressure was mainly raised by participants whose parents were becoming much older, or who were raised by their grandparents, who would beg them to fulfil their wishes. In addition, some parents conspired with religious leaders who would scorn and admonish their daughters for prioritising a career over marriage.

It also emerged that in some families, female siblings are expected to get married according  to birth order. One Christian participant noted, based on experience and observation, that in her home context,^4^ a woman with younger siblings who prioritises career establishment over marriage becomes seen as a ‘nuisance’ to her parents and extended family members who would keep nagging her to *“open the marriage doors for the rest of the siblings*” (IDI, Female, #32, MCR, married). She also felt internal pressure to conform to such expectations based on her religious beliefs. Similarly, a Muslim male participant emphasised that based on his cultural and religious beliefs, “*women and men can only get blessings in life once they are married… It is something you can’t run away from as it’s a rite of passage”* (IDI, Male, #16, MSc, unmarried).

Some unmarried male researchers also reported experiences of shame and ridicule from parents, siblings, and peers in home communities, who could not understand why “*you are only prioritising getting degrees over marriage and establishing family”* (IDI, Male, #20, PhD). They faced occasional demeaning statements such as “*life seems to be slow for you*” (IDI, Male, #08, PhD) and “*you will die in school”* (IDI, Male, #19, PhD).

Overall, most participants explained family members and broader society including friends, had limited understanding of the requirements and importance of scientific work and are thus not sympathetic to the dilemmas facing them in establishing their careers. Notably, most scientific researchers who participated in this study (46/58) identified themselves as the first highly educated generation in their family and the first to pursue such a career.

### Theme 3: Navigating ‘two different lives’

This theme focuses on how gender and other identities shape researchers’ everyday experiences of navigating the ‘two different lives’ of career and family, and the resultant implications for their career progression and personal well-being.

#### Time pressure and sense of ‘work-life’ imbalance related to scientific writing

*Time pressure particularly disadvantages women in career progression*: Although scientific writing and publication was considered essential for upward career mobility, making time for this ‘activity’ was perceived as ‘difficult’ by everyone. However, most participants (both female and male) expressed that it is particularly challenging for women given the unequal gender division of labour within the family, which renders women—regardless of marital or parental status—‘time poor’:

Even getting time to sit and write just becomes so difficult…So it is not easy. I just feel we [women] are ‘time poor’…we struggle to write and manage other major family responsibilities [elderly care]… of course moving to the next level for scientists, it is always about publishing (IDI, Female, #27, PDF, unmarried).

Men were perceived as ‘privileged’ with the opportunity of staying in the office for longer working hours compared with women who must leave earlier to fulfil family responsibilities:

I have to pull my weight just as much as the male counterpart who sits next to me can pull their weight… these guys [men] stay in the office until 9 pm and for me I have to leave at 3 pm to pick children from school…help them with homework…cook dinner and ensure they are in bed by 8 pm…then I continue working maybe until 11 pm and wake up by 5am to repeat it all over again… I am trying to manage ‘two different lives’ which is a struggle for me (IDI, Female, #26, PDF, married, under 5-year-old children).

The above participant further expressed that a married woman staying in the office till late means the family suffers and society will judge her as a woman who failed to manage her home. This places women at a disadvantage in terms of their opportunities to allocate and spend the ‘extra time’ required to meet institutional requirements for career progression. Some men also commented on the impact of this on women’s career progression opportunities; for example: *“I always wonder how women make it in science beside managing their childbearing and family responsibilities” (IDI, Male, #05, PDF, married, under 5-year-old-children).*

*Time pressure impacts differently on women and men*: Most female researchers, at all career levels irrespective of their marital and parental status, reported that there is no work-life balance in science careers, which often meant long hours working both at home and at work. Some asserted that for women to achieve career progression, they have to work much harder compared with men at fulfilling their dual responsibilities. Concerns about continued poor ‘work-life balance’ leads junior and early career female researchers, to question whether to stay in a scientific research career:

Time is a big one…For me being able to balance between your family and work is a big challenge…that has been going through my mind lately. I definitely like science, but I am beginning to question myself of whether or not I want to stay in science if it continues to be the way it is (IDI, Female, #18, MSc, unmarried).

Most men perceived themselves as better off compared with women in terms of time pressure. Some male researchers also shared concerns about ‘poor work-life balance’, particularly at doctoral and post-doctoral career stages. However, their explanations revolved around the long-hours working culture of science which puts a toll on their ‘time’ allocation for social activities and personal life. Thus, they might have an advantage in career progression, but at a personal cost. For this reason, some already felt they were unlikely to continue with such a career path:

The reality is that I don’t seem to have the ‘right’ work-life balance… it’s difficult. I am [at work] at night and on weekends…it takes up a lot of ‘time’ …[and] has had a toll on my social life … you have to keep off a number of things… Actually, I feel like I need to change … to find the kind of work that gives me the flexibility for personal time … I can’t continue working this way for the coming years (IDI, Male, #10, PhD, unmarried).

*Additional time demands for language minority research scientists*: A unique challenge elucidated by participants who identified themselves as language minorities, was the additional time requirements to read and write in English. Participants from Francophone countries explained that, *“As Francophone fellows, we have no choice but rather you must put in effort to learn English, which is a universal language of scientific communication”* (IDI, Male, #04, PDF). Many of them saw this as a challenge that they were determined to meet to ‘prove themselves’ equally as good research scientists as the Anglophones. However, preparing and delivering scientific outputs and presentations in English requires additional time allocation:

We still need more ‘time’ for us to write in English…most of the time we write first in French then we translate into English …[then] share the text with the people from English speaking countries to correct every text…[also]when making [a] presentation, you need first to prepare your talk,… present it to them…[so that] they correct it before the meeting. The whole process takes much ‘time’…that is my greatest challenge (IDI, Male, #01, Francophone PDF).

Female Francophone researchers are thus doubly disadvantaged by the additional time burden of writing in English as well as their domestic responsibilities:

The language barrier is a very big problem for us…writing becomes a bit more difficult when you have kids, [as] you have more duties and you have to share your ‘time’ in between family and writing. So of course, it impacts to the ‘time’ you have to dedicate to grant writing or publication writing…So it takes a long ‘time’ for us (IDI, Female, #04, Francophone PDF).

#### Pressures around participation in scientific mobility-oriented ‘activities’

The expectations of geographic mobility oriented ‘activities’ required to progress in a scientific career emerged as a specific dimension that differentially affects women and men in ways that are gendered and intersect with other aspects of their identities. All participants narrated that the DELTAS Africa initiative offered them travel grants for attending and presenting at conferences as well as undertaking exchange programmes through visiting research collaborators in the global north. Such visits vary in duration across different consortia and career stages, but tend to last between three to six months. However, even though such opportunities were equally presented to them, some women and men researchers experienced barriers to uptake, which were shaped by their marital and parental status, nature of partnership, and positional hierarchy in the family.

***Women’s experiences***: Some women researchers with young children, whether they were married or not, explained that their ability to take up these opportunities was limited by their socially prescribed childbearing and care responsibilities. They tended to be selective of which travels to pursue and to shy away from those that required staying away for a longer period in favour of fulfilling their caring responsibilities. One participant contrasted this with her male colleagues’ ability to take up all the opportunities available:

For us women, you can't force it to happen, but rather choose which event to attend while men can decide to go for all events” (IDI, Female, #25, PDF, married, under 5-year-old children).

Another explained that childbearing had ‘slowed down’ her career for this reason:

This is a woman’s life, so it is challenging for every woman who has children. There are occasions where I have failed attending conferences or travelling abroad for an important training…either I was pregnant, or I had a very young breastfeeding baby…so it can slow down some steps in establishing your career niche (IDI, Female, #05, PDF, married, under 5-year-old children).

Male participants agreed with this; for example:

For a woman, if you have children who are less than one-year-old, it is not easy to go [abroad] for three months. Perhaps you have to go with your children if it is possible…I think that is the good thing that we [the programme] can do (IDI, Male, #03, PDF, married, under 5-year-old-children).

Notably, despite their reliance on employing house-helps for childcare while at work, most mothers were apprehensive of leaving their children under their care while on travel for fear of child abuse.

This problem was not only perceived as that of physically taking care of children but also a more normative symbolic importance of always being ‘available’ and having a primary focus on mothering responsibilities. Whilst both married and unmarried female researchers with young children sought childcare support, which is a vital *‘resource’*, from the extended family, they expressed frustration that this was sometimes given grudgingly. In-laws may remind them indirectly of the primacy of their caring duties with statements such as: *“remember you have duties, don’t abandon them” (*IDI, Female, #27, PDF). Some highlighted that even if childcare support during travel is provided by the programme, sometimes the in-laws and extended family members would question “*why you leave the family behind and often fail to participate in the family events*” (IDI, Female, #22, PhD, married, under 5-year-old-child). This participant narrated that continuous failure to do so could result in marital discord and break-up, leading to emotional suffering, a view that was consistently shared by other married female participants. One participant narrated her own experience of this:

So it became stressful …my partner [non-dual career couple] could not understand why you are not ‘available’…you are always on travels… He thinks you are going beyond what he understood you as a woman. …. Yeah, ‘relationships’ went through the roof! That one [laughs], I mean it is very hard for us women (IDI, Female, #31, MCR).

Similarly, while making reference to the normative symbolic requirements of constant ‘availability’ in most socio-cultural contexts in Africa, a male participant explained that:

In our African culture in general, women are not allowed to travel all the time. For men it’s normal as they are breadwinners and thus obliged to travel and fend for their families …not the contrary. …The society doesn’t have a problem with men travelling compared to women because they see you are working and trying to provide for your family…it is essential…They [men] are barely questioned when they stay away from home for long (IDI, Male, #02, PDF).

***Men’s experiences***: Notwithstanding the stereotypical normative assumption that men are breadwinners, some married men researchers feared and declined to undertake the long-term travels expected in exchange programmes. They perceived this a as a compromise due to the normative expectations of always being ‘available’ at home with the rest of immediate family members specific to their situation. For instance, a participant feared being criticised by his nuclear and extended family for “*moving out too much….[as] they have heard stories of people [men in the community] who when they go out [abroad], some of their marriages come to an end*” (IDI, Male, #10, PhD, married, under 5-year old-children). This was based on the perception that such men are likely to establish another family abroad. He further explained that such concerns led to a lag in acquiring the necessary scientific skills required to enable him to carry out his research work.

In another instance, a married male researcher, who had additional responsibilities as a de facto household head of his extended family following his father’s death, recounted how the normative symbolic importance of constant ‘availability’ for the family inhibits his participation in scientific mobility. He narrated that by virtue of being the only son from his nuclear family, he bears the customary responsibility to preside over important family social events such as marriage ceremonies and death of a family member; in his absence such matters get postponed, which makes him feel he is a nuisance to his family. He expressed that taking time off work for these responsibilities can also be interpreted by supervisors as showing a lack of commitment to work. He expressed that sometimes men like himself “suffer in silence” over these dilemmas, explaining: “*Sometimes, we don’t say certain things!*” (IDI, Male, #23, other identities withheld). Consequently, “*such customary family obligations [which] dictates that as a man you need to be home taking care of such issues…can weigh down on your career as they come with a lot of stress…[which can] pull you back a lot [from progressing]*” (IDI, Male, #23, other identities withheld). Thus, some male researchers experience either negative effects on their career progression or their personal well-being due to navigating these competing pressures.

#### Scientific mobility challenges exacerbated for dual scientific career couples:

Participants who were married to another scientist with young children cited scientific mobility as their greatest challenge to progression, particularly when their travel dates coincide. They had to make decisions about who should travel, with some explaining that they followed a rotational travel plan as “*there is no proper formula for resolving the child-care puzzle when that happens*” (IDI, Male, #25, PDF). In this situation, female participants in such unions shared their personal frustrations about not only limiting their own travel, but also bearing the brunt of childcare responsibilities alone when their partner travels, affecting their career progression:

I bear the brunt of everyday care of dropping and picking them (children) from school and caring for them once they get home, which is affecting my progress with doctoral studies…sometimes you have to put those travels on hold…you would want your partner to be supporting such endeavours but unfortunately you are just alone (because of geographical separation) (IDI, Female, #14, PhD, dual scientific career couple, under 5-year old-children).

The above participant expressed thoughts of quitting scientific research in pursuit of clinical practice. Other female researchers in such a marital union explained that the problem extended beyond the practical problem of childcare to the normative expectation of their availability and responsibility: *“If both of you are out, whatever happens to the children in Africa, the woman is definitely blamed…so most women like myself don’t bother taking them up”* (IDI, Female, #11, PhD, dual scientific career couple).

### Theme 4: Potential strategies utilised by women for navigating the ‘two different lives’

Both female and male participants observed that the timing of the performance and establishment of one’s scientific research career, mainly happens while in their 30s, the period during which most women researchers experience the highest level of career interruptions because of childbearing and rearing responsibilities. This puts them at a disadvantage in terms of achieving the milestones within a normative career path as compared with men:

Sometimes depending on the age, it becomes very difficult for women to build their careers…women who are generally my age [mid 30s] have young families…This is the point at which they now feel they can establish their career which creates a huge conflict [and] poses a challenge for them as they have to either take a break from research or their career progression to bring up their family (IDI, Male, #26, PDF, married, under 5-year old-children).

In the context of the challenges described above, many women researchers made different and conscious trade-offs between their ‘time’ commitments for family and scientific research activities. In narrating this, and their considerations in making these trade-offs, they used several key metaphors such as the *‘biological clock and career clock’*, the ‘*glass ball and rubber ball*’, and the concept of ‘*sacrifice*’. Male researchers did not speak about such strategies.

The metaphor of the ‘biological vs career clock’ pitted the idea of a ‘ticking’ ‘biological clock’—a limited window for fertility—against a ‘career clock’, which denoted a steady focus of establishing oneself career-wise, expressing the sense of time pressure. The time pressure of the career clock was described by both female and male participants as increasing with seniority, as expressed by a female participant as follows:

As you move up, it becomes harder and harder demanding much time, energy and attention. […] the family life is one of the major competing interest, and unfortunately the burden always lies with the woman… You are constantly split between managing these two things …That is why there are a lot more girls doing PhDs and then when it reaches post doc all of them will tell you, I can’t take the pressure of science (IDI, Female, #29, MCR).

Women attempting to ‘chase’ both ‘biological and career clock’ complained of ‘*mental slowness*’ and constant fatigue which they felt contributed to slowing down their career advancement compared with their male counterparts.

Others articulated that in life, women are presented with two balls: a ‘*glass ball’* and a *‘rubber ball’*. The *‘glass ball’* denoted the normative expectation to get married and establish a family, which when dropped, is difficult to recover as it will be broken completely. The ‘*rubber ball’* denoted the career itself, which when dropped, will keep bouncing—that is you can always have it back—expressing the idea that one’s personal life is more fragile than a career and needs to be protected where one is unable to effectively ‘juggle’ the two balls.

Many female participants narrated a sense of making ‘*sacrifices’,* either of their career progression in favour of their personal and family life or vice versa. One woman expressed this as follows: “*as a woman you cannot throw your children and husband on the street because of career progression”* (IDI, Female, #12, PhD). This participant, who was in a dual scientific career marital union, narrated how she had taken a career break from science by taking up an administrative job for close to ten years which enabled her to raise her children. She later resumed her science career (catching back the ‘*rubber ball’*) by taking up a DELTAS research fellowship. She emphasised that achieving certain milestones by a certain age as is normative in scientific career path can be difficult especially when age is used as a criterion for selection as well as good publication and grant record. She expressed that this amounts to a form of discrimination, along with employers being unwilling to make allowances for such career breaks in recruitment. Consequently, she observed that such women can end up getting ‘stuck’ in lower-level scientific research positions or opt out of this career either part way through to senior level or in early stages of the ‘pathway’. Those women who narrated prioritising their ‘career clock’ explained that their relationships have suffered. For example:

Our relationship just ended like that…he thought I am busy chasing this career by not thinking about settling down for marriage…he gave up with me. I have been suffering in silence since then [for the past 2.5 years] … it is difficult… I really don’t want to speak about it at length, it is hard (IDI, Female, #06, PDF, unmarried).

Some junior and early career women researchers who were already married or were planning to get married and establish families expressed that they had very few examples of women in senior scientific positions who are also in successful marriages. Their perception, that most senior women had to ‘sacrifice’ their marriages to enable them progress in their careers, negatively impacted their potential ambitions for career progression in scientific research. Overall, most women, especially at junior and early career stages, regardless of their marital and parental status, viewed an academic scientific research career as ‘a huge battle’. This seemed unappealing in view of the ‘sacrifices’ they felt they would need to make for these careers:

It’s a ‘huge battle’ for women which creates difficulties for them to just make a decision on whether to progress to next level in their science career or not… So, you are going to worry about the impact that that decision is going to have to the rest of your family (IDI, Female, #13, PhD, married, under 5-year old child).

Some were already considering alternative career pathways, including: academic teaching roles and pursuing research consultancies on the side; research and grant management; or developmental non-governmental organizations. They perceived such opportunities as likely to enable them to achieve a better work-life balance. In the same vein, a male participant argued that even though the overall DELTAS programme has almost achieved gender parity in recruiting female and male researchers, women face greater barriers to progression as *“…it’s a steeper hill for women to climb on …which requires much ‘sacrifice’”* (IDI, Male, #09, PhD).

## Discussion

This study has contributed towards illuminating the underlying familial and socio-cultural drivers of intersectional gender inequities, and how they interact in shaping experiences and career progression of researchers as they engage in their day-to-day scientific research ‘activities’. Our findings show that advancement in scientific research careers require extensive ‘time’ commitment, a crucial ‘resource’, to meet normative requirements of long working hours with occasional scientific mobility. Such requirements interact with gender divisions of labour and normative societal expectations at family level, in ways that differ for women and men and are further shaped by other aspects of their identities. The unequal gender division of labour in the family often reduces women’s opportunities to compete with men in terms of time availability to commit to scientific research. This creates dilemmas around whether to ‘sacrifice’ career or family or negative impacts on well-being through efforts to pursue both. These pressures are particularly acute for women due to the expectations to establish both careers and families during the same period. All participants agreed that this systematically disadvantages women in career progression, contributing towards their underrepresentation and attrition in scientific research careers. Francophone women and men in SSA experience further disadvantage due to the additional time burden of ‘translating’ between French and English. However, the normative gender roles as family heads also create pressures and dilemmas for some men. For instance, the expectation of them as ‘breadwinners’ has a negative impact on their well-being and opportunities for ‘work-life balance’, including contributions to their family lives. Neither are opportunities equal between men, with Francophone speakers facing clear disadvantage.

### Normative scientific career progression as inherent in western capitalist system

The normative scientific career progression described by participants is structured around the idea of spending ‘extra time’ at work, which does not reflect social realities for most women and some men in SSA. Such expectations embody the prevailing western capitalist scientific system of long hours-culture through which the ‘ideal scientist’ is defined as an individual with unlimited time commitment to science throughout their entire working life [33]. As reflected in our findings, capitalism interacts with patriarchy to create an inherent bias against female scientists with caring and other domestic responsibilities, who are disproportionately penalised and pushed out of the system [33, 34]. This is particularly due to the bottleneck created by conflicting time requirements during reproductive years [3]. However, this intersection between patriarchy and capitalist extraction of value in scientific labour also reduces men’s opportunities to contribute to and benefit from family life as well as pursue other creative interests [3].

#### The impact of gender roles and social norms on equitable scientific career progression

In line with a number of other researchers in SSA [4, 6, 9, 10, 35], this study found that women’s socially ascribed caring responsibilities and expectations of marriage within the ‘rules’ of the family as an institution are strongly reinforced by familial and wider societal pressure [10]. Our findings support others which have shown that some women interrupt their careers due to adhere to social norms of marriage and establishing family, lest they be ostracized and blamed for neglecting them [6, 9, 10, 36]. To this we have added the new insight that birth order can exacerbate the pressure to marry at a particular time for some women. Further, we found that a generational educational gap compounds this problem for many women and some men as many participants in this study were the first in their family to pursue a scientific career path, reducing familial understanding of its particular pressures.

We also recognise that employing web technology could help alleviate some of the difficulties experienced by some women and men researchers who face constraints to participate in scientific-mobility oriented activities. Teleconferencing and webinars could enable researchers at all career stages to present aspects of their work to others, as well as attend online trainings without requiring to travel great distances [37]. Miller and colleagues [38] quantitatively examined the impact of the internet on the research careers of female scientists in SSA and South Asia, who found that the introduction of the internet is widely expected to diminish professional women’s constraints with physical mobility, thus could aid in reducing gender differentials resulting from this. Such a shift may potentially be accelerated by increases in online conferencing driven by the COVID-19 pandemic, but this has yet to be evidenced.

#### Nature of childcare support received from the family

This study also presents new insights on the challenges related to marital and parental status by indicating that these are not just practical challenges around provision of childcare but also symbolic ones around normative ‘availability’ and expectations of women to prioritise families rather than careers. In a recent study, Khisa et al. [39] have highlighted the positive outcome of supporting women’s practical gender needs around childbearing and caring through financially supporting husbands to travel with their spouse to provide childcare during short and long-term travel. The authors contend that such efforts are beginning to pay off through men/fathers playing a more active role in childcare even on return. Consequently such an initiative may support shifting the patriarchal gendered institution of family to be more accommodating to women to successfully pursue careers in science research [39]. However, how this might play out for dual and non-dual scientific career couples remains unexplored.

#### Francophone researchers as disadvantaged scientists

An intersectional perspective has shown that language minority status also emerged as an additional layer of disadvantage, further amplifying the ‘time’ pressure for Francophone women and men researchers in meeting the expectations of scientific research ‘activities’. However, women Francophone speakers with caring responsibilities emerged as particularly disadvantaged. Various scholars [40, 41] have observed that as a pre-requisite for career progression, it is increasingly becoming a requirement for researchers to publish their work in English language journals. This is to enable them gain visibility to the wider audience and international recognition, which is a struggle for many non-Anglophone speaking scientists. We argue that placing emphasis on English as a standard language for scientific research communication is a form of neo-colonialism that minimises the presentation of scientific research from other languages. This inadvertently limits wide dissemination of research results by researchers from such disadvantaged language minority populations [42], thus reducing their research productivity as well as visibility and research collaborations.

#### Experiences of men researchers and dual scientific career couples

By going beyond a ‘women-only’ perspective, which has been the trend in most studies [20] towards consideration of men’s experiences, this study provides new insights on the challenges faced by some men. These are produced by the intersection of institutional scientific career norms and their gender identities, particularly where their positional hierarchy in the family creates additional family responsibilities due to customary values and expectations that require their frequent ‘availability’ at home. We further established that the particular challenges faced by dual career couples with young children regarding scientific mobility. This has been observed in European settings [43], but not to date in the SSA literature. Our findings concur with existing studies suggesting that among such couples, the female partner is more likely to forfeit travel ‘activities’ [43] with negative implications for her career, due to lack of childcare availability. However, the challenges faced by men suggest that the gendered assumptions of constant availability underlying the scientific career model may be inimical to personal and social well-being for both sexes [33]. Therefore, unless inequalities in the opportunity of individuals to meet expectations of unlimited time availability to devote to scientific research are considered a structural problem rather than an individual problem, they are likely to persist.

#### Trade-offs and resultant costs of managing the ‘two different lives’

This study provides new insights on how women make decisions about whether to spend additional time on work, particularly during the time period of intense demands of young families. This has consequences for their career progression, their individual well-being and their social relationships. Most women feel this acutely as a dilemma between managing ‘two different lives’, and use various metaphors to discuss the strategies utilised and the ‘trade-offs’ involved. The ‘biological clock’ and ‘career clock’ metaphor was also used by participants in another qualitative study in SSA [10]. The ‘glass and rubber balls’ metaphor has not been identified in previous studies in SSA but emerged as a common figure of speech from a qualitative study conducted amongst women from eight Arab Middle Eastern countries^5^ [44]. These metaphors, and the narratives of our participants emphasise the high social costs to women of pursuing scientific careers, including divorce and separation or opting not to marry, in line with some studies in Europe [33, 45]. This was underscored in our study by early career female participants pointing to the lack of positive role models who had succeeded in their careers without ‘sacrificing’ marriage and family. Those women in our study who attempted to meet both familial and career expectations reported exhaustion. Some of our participants narrated emotional suffering that was rarely expressed in their daily lives. Thus, all these strategies have consequences for either women’s well-being or their lower representation in scientific careers, especially in more senior positions.

Our study also found that even though men generally did not talk about dilemmas about managing the ‘two different lives’, some did express a sense of the social cost to their well-being and social relationships of spending the ‘extra’ time at work. They experienced a sense of work-life imbalance that was detrimental to their well-being, with some indicating their likelihood of opting out of scientific research career, citing a lack of time for leisure. It is note-worthy that female researchers did not even refer to perceived needs for leisure, which likely reflects gendered norms and expectations, especially for women with caring responsibilities. Some scholars have argued for a fundamental rethinking of current scientific research and family systems for the benefit of all [46]. Whilst such a rethink may particularly benefit women in terms of well-being (including the opportunity for leisure time) and career progression, our study suggests that such a rethink would also offer benefits to male researchers’ well-being, contributing to the retention of male scientists.

The empirical findings of this study generally support the conceptual framework posited based on existing literature and relevant models of career progression and social relations [16] and has contributed new insights about how intersecting aspects of individual identities create particular pathways in specific contexts. This analysis has provided new insights into often-overlooked types of identities such as dual career couple and individual’s positional hierarchy in the family, in shaping inequities in career progression.

#### Policy and practice implications

The results of this study indicate that fostering equitable scientific research career progression for women and men in SSA requires understanding, recognising and taking actions to address familial and societal drivers of intersectional gender inequities in order to reduce career disadvantage and improve well-being of researchers. A gender-transformative approach is required, which goes beyond steps to ameliorate the impact of unequal gendered power relations to transforming them [47]. This will require sustained action both within and beyond scientific research institutions and funders. However, concrete policy and practice measures and approaches that can be taken by employers and funders include:

First, reforms in institutional human resources policies and systems to provide researchers with more practical support to parenting such as childcare and parental leave, including for men. This may help women to navigate the ‘two different lives’ and also to shift norms so that this work is less seen as the sole responsibility of women [39, 48]. Removing age-related expectations from hiring/promotions policies is also important. In addition, promoting workplace practices and incentives for healthy work-life balance for all, for instance through active discouragement of overworking, should be considered.

Second, there is a need for a more fundamental re-think of the normative scientific career structure to create equitable opportunities, improve diversity, and also the well-being of both female and male employees. The science leaders and grant awarding bodies have a role to play in redefining the adoption in SSA of a western scientific research system that prioritises research productivity, which does not account for the social realities of African researchers. Attention should be paid to developing a more locally appropriate and achievable approach to measuring ‘excellence’ for individuals, in line with existing debates at the level of national scientific research funding systems [49]. This may include considering research achievements in the context of research opportunities for individuals (e.g. over a full time working equivalent period) [50]. Additionally, there is a need for greater flexibility in setting age limits as a criterion for eligibility for fellowship and other research appointments. Tackling the expectation of long working hours is also required. This is a cultural change but will also need a revised expectation of productivity within given working hours. Support is required from SSA governments as local funders and in setting expectations with external funders.

Third, additional support and potential adjustments to expectations for language minorities in science across SSA, including but not limited to Francophone speakers, is also required to create equality of opportunity. This could be achieved through enhanced institutional collaborations between Anglophone and Francophone researchers to help strengthen researcher English language skills. However, re-shaping local scientific eco-systems may also require attention to the role of publications and grant submissions in languages beyond English. External research funders should also consider granting additional time or support for submission to non-English language speakers.

#### Study limitations

Findings from this study should be considered in light of the following limitations. First while the integrated conceptual framework highlights the intersection of gender and physical disability, we were not able to identify researchers who identified as disabled within the sampled consortia and the overall DELTAS Africa initiative. Efforts to identify and recruit such individuals from the wider host and participating institutions in selected consortia were prevented by the need for country-level ethical clearances for each institution. This was not possible within the time constraints of the study. Second, participant concerns about anonymity and confidentiality prevented the presentation of nuanced comparisons with regard to nationality and ethnicity. Third, we acknowledge the underrepresentation of female PDFs in our sample. This was not because of study design. Despite significant follow up efforts, we experienced lower take up of interview offers by female PDFs. Despite these limitations, we contend that this study provides useful information upon which to understand the issues and begin to address the gendered familial and socio-cultural drivers of challenges facing research scientists in SSA.

## Conclusions

The findings presented in this paper reflects the experiences of women and men scientific researchers at various career stages characterised by multiple social identities in three purposively sampled consortia within the DELTAS Africa research capacity strengthening initiative. This study is the first of its kind to demonstrate how intersectional gender analysis through use of qualitative research methods may advance novel ways of understanding the differential hidden familial and socio-cultural challenges that contribute to inequitable scientific career progression. Specifically, we have shown the importance of considering multiple social identities such as age, marital status, parental status, presence of dependants, positional hierarchy within family, nature of partnership (dual and non-dual career couple) and patriarchy and capitalism as systems of power, oppression and privilege in shaping inequities in career progression. It is important to take into consideration the fluidity of individual social identities, which contributes to slow progression and the loss of researchers along the scientific research pathway at different career stages when their identities change. A fundamental re-think of the normative scientific career structure in SSA is required to create equitable opportunities, increase diversity and improve the well-being of both female and male scientific researchers.

## Data Availability

Individual privacy could be comprised if data is made publicly available. For this reason, data cannot be shared.

## Abbreviations

DELTAS Africa: Developing Excellence in Leadership, Training and Science in Africa
DELTAS ARC: DELTAS Africa research consortia
IDI: In-depth interview
MCR: Mid-career research scientist
MSc: Masters
PDF: Post-doctoral research fellow
PhD: Doctoral researcher
SSA: Sub-Saharan Africa

## Footnotes

1 https://www.aasciences.africa/sites/default/files/Publications/DELTAS%20Africa%20factsheet_2018_0.pdf site accessed on 6th January 2020.

2 In this study, we define such partnership as non-dual career couples.

3 Short-term study or placements were most common in the DELTAS ARC.

4 Identity of the ethnic community and country withheld for purposes of confidentiality as requested by the study participant.

5 These countries included: Egypt, Jordan, Saudi Arabia, Kuwait, Lebanon, Oman, Palestine, and Qatar.
